# Can Intestinal Pseudo-Obstruction Drive Recurrent Stroke-Like Episodes in Late-Onset MELAS Syndrome? A Case Report and Review of the Literature

**DOI:** 10.3389/fneur.2019.00038

**Published:** 2019-01-31

**Authors:** Delia Gagliardi, Eleonora Mauri, Francesca Magri, Daniele Velardo, Megi Meneri, Elena Abati, Roberta Brusa, Irene Faravelli, Daniela Piga, Dario Ronchi, Fabio Triulzi, Lorenzo Peverelli, Monica Sciacco, Nereo Bresolin, Giacomo Pietro Comi, Stefania Corti, Alessandra Govoni

**Affiliations:** ^1^Neuroscience Section, Department of Pathophysiology and Transplantation (DEPT), Dino Ferrari Centre, University of Milan, Milan, Italy; ^2^Neurology Unit, IRCCS Foundation Ca' Granda Ospedale Maggiore Policlinico, Milan, Italy; ^3^Neuroradiology Unit, Fondazione IRCCS Ca' Granda Ospedale Maggiore Policlinico, Milan, Italy; ^4^Neuromuscular and Rare Disease Unit, Department of Neuroscience, Foundation IRCCS Ca' Granda Ospedale Maggiore Policlinico, University of Milan, Milan, Italy; ^5^Neurology Unit, Neuroscience Section, Department of Pathophysiology and Transplantation (DEPT), Dino Ferrari Centre, University of Milan, IRCCS Foundation Ca' Granda Ospedale Maggiore Policlinico, Milan, Italy

**Keywords:** MELAS, mitochondrial disorders, intestinal pseudo-obstruction, stroke-like episodes, gastrointestinal disturbance

## Abstract

Mitochondrial encephalomyopathy, lactic acidosis, and stroke-like episodes (MELAS) syndrome is a maternally inherited mitochondrial disorder that is most commonly caused by the m. 3243A>G mutation in the MT-TL1 mitochondrial DNA gene, resulting in impairment of mitochondrial energy metabolism. Although childhood is the typical age of onset, a small fraction (1–6%) of individuals manifest the disease after 40 years of age and usually have a less aggressive disease course. The clinical manifestations are variable and mainly depend on the degree of heteroplasmy in the patient's tissues and organs. They include muscle weakness, diabetes, lactic acidemia, gastrointestinal disturbances, and stroke-like episodes, which are the most commonly observed symptom. We describe the case of a 50-year-old male patient who presented with relapsing intestinal pseudo-obstruction (IPO) episodes, which led to a late diagnosis of MELAS. After diagnosis, he presented several stroke-like episodes in a short time period and developed a rapidly progressive cognitive decline, which unfortunately resulted in his death. We describe the variable clinical manifestations of MELAS syndrome in this atypical and relatively old patient, with a focus on paralytic ileus and stroke-like episodes; the first symptom may have driven the others, leading to a relentless decline. Moreover, we provide a brief revision of previous reports of IPO occurrence in MELAS patients with the m.3243A>G mutation, and we investigate its relationship with stroke-like episodes. Our findings underscore the importance of recognizing gastrointestinal disturbance to prevent neurological comorbidities.

## Introduction

Mitochondrial encephalomyopathy, lactic acidosis, and stroke-like episodes (MELAS) syndrome is one of the most common maternally inherited mitochondrial disorders, with an estimated prevalence of 0.2:100,000 in Japan (1). MELAS was first described by Pavlakis in 1984 (2). The most common genetic defect is an adenine to guanine transition at position 3243 on the mitochondrial DNA in the MT-TL1 gene, which encodes the transfer RNA (tRNA)^Leu(UUR)^. The prevalence of this mutation was found to be higher than previously reported, with a prevalence of 0.24% in a large Caucasian population (236 out of 100000 persons) (3). The m.3243A>G mutation results in altered mitochondrial translation and protein synthesis, which affect the electron transport chain (ETC) complex subunit structure and lead to impaired mitochondrial energy production (4).

Defects in mitochondrial metabolism lead to a disparity between dysfunction in energy generation and energy demand from organs and tissue. Therefore, the signs and symptoms are extremely variable and mostly involve organs with higher energy requirements that are particularly vulnerable to an insufficient energy supply, such as the nervous system and the skeletal and cardiac muscles. Nevertheless, given the heteroplasmy phenomenon (i.e., different tissues and organs harbor different amounts of mutant mtDNA, since the mutation is not present in all copies of the mtDNA, and the mutant mtDNA is randomly distributed among daughter cells during cell division), clinical manifestations are variable among different individuals and among different tissues in the same patient.

Stroke-like episodes are paradigmatic symptoms of MELAS. These symptoms occur in almost all affected individuals and represent a common initial clinical manifestation. They manifest with an acute onset neurological deficit that is sometimes accompanied by headache, vomiting, and seizures. Other common signs and symptoms of the disease are represented by muscle weakness, exercise intolerance, diabetes, lactic acidemia, dementia, and gastrointestinal disturbances.

Intestinal pseudo-obstruction (IPO) is most commonly observed in mitochondrial neurogastrointestinal encephalomyopathy (MNGIE), although this symptom can also affect patients with MELAS syndrome (5), particularly those with the m.3243A>G mutation; however, IPO remains an underrecognized condition. IPO is a rare and severe condition that is characterized by abdominal distension, nausea, vomiting, and pain due to impaired propulsive activity in the gastrointestinal tract and resembles mechanical intestinal obstruction (6). The pathophysiological mechanisms underlying IPO in mitochondrial disorders are most likely related to mitochondrial dysfunction and energy metabolism imbalance, similar to the situation in the brain during stroke-like episodes. The importance of this condition has been identified recently, since it can represent a prognostic factor in m.3243A>G mutated patients (7).

We describe the case of a 50-year-old male patient who started to develop ideomotor decline and relapsing episodes of abdominal distension, pain, and vomiting after an ischemic stroke, which were consistent with a diagnosis of IPO. Brain magnetic resonance imaging (MRI), magnetic resonance spectroscopy (MRS), muscle biopsy and genetic analysis led us to reconsider the previous ischemic stroke as a stroke-like episode and revealed a late diagnosis of MELAS syndrome caused by the m.3243A>G mutation. After diagnosis, several stroke-like episodes occurred in a short time period, leading to a rapidly progressive cognitive decline and finally to the patient's death.

Although the adult form of MELAS syndrome is thought to display a more favorable course, our patient exhibited an aggressive and rapidly progressing deterioration. Gastrointestinal disturbance and stroke-like episodes share common pathogenic mechanisms (i.e., dysfunction in mitochondrial energy production); one symptom may precipitate the other, triggering a cascade of stroke-like episodes and leading to metabolic failure in the patient. Given the late clinical presentation, presence of gastrointestinal dysmotility and peculiar clinical course, our patient can provide interesting insights into both the pathophysiology and management of mitochondrial diseases. In particular, earlier detection of gastroenterological disturbances in these patients may prompt earlier identification of neurological comorbidities, leading to appropriate therapeutic strategies and improved clinical outcomes.

## Case report

A 50-year-old man received medical attention due to subacute onset of relapsing intestinal subocclusion episodes characterized by vomiting, diarrhea and marked abdominal distension, which gradually developed within approximately 40 days.

**Figure 1:**
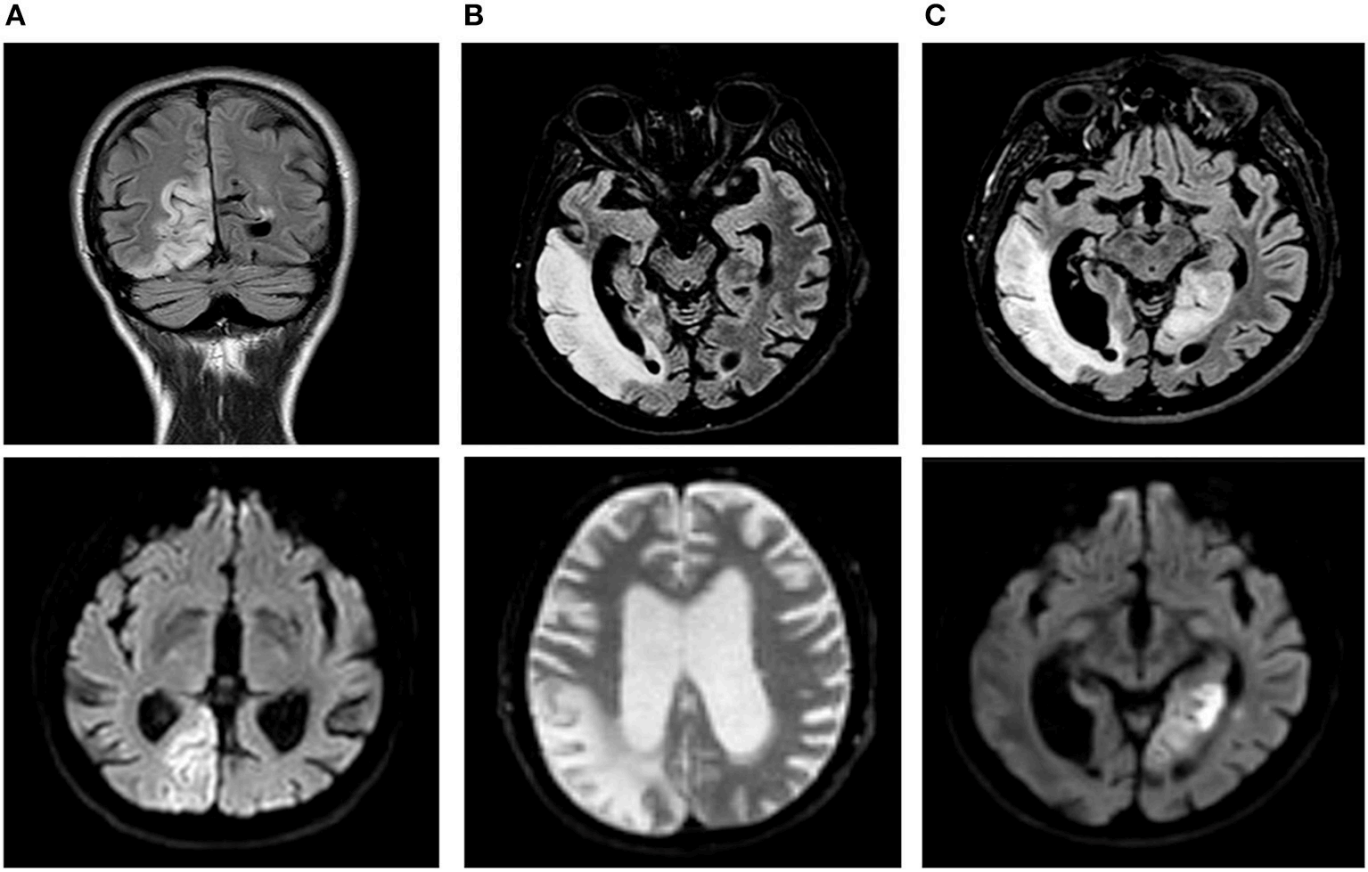
Temporal evolution of brain magnetic resonance imaging (MRI). FLAIR (fluid attenuated recovery) sequences are shown in the upper line, and DWI (diffusion-weighted imaging) sequences are shown in the lower line. **(A)** First stroke-like episode initially interpreted as an ischemic stroke. A high signal occurred in the diffusion-weighted imaging (DWI) sequences in the right temporo-occipital lobes and the corresponding fluid attenuated recovery (FLAIR) sequence. **(B)** Second stroke-like episode: three months later, evidence of a wider lesion was found in the right temporal, parietal and occipital lobes together with initial enlargement of the ventricular spaces. **(C)** Third stroke-like episode: a new cortical DWI abnormality was observed in the left medial temporal and occipital lobes; marked and diffuse atrophy was found in the brain parenchyma.

His past medical history included dyslipidemia and previous lipoma removal; additionally, his relatives reported apathy, loss of interest in work and hobbies and progressive social isolation occurring over the last two years. Two months before his presentation, he was hospitalized for acute onset of blurred vision and phosphenes in the left visual field that were associated with a frontal headache and confusion. The neurological evaluation revealed left hemianopia, temporal, and spatial disorientation and moderate psychic and motion slowness. Brain computed tomography (CT) and MRI showed a right temporo-occipital lesion with a high signal in the diffusion-weighted imaging (DWI) sequences, which was interpreted as an ischemic stroke (Figure 1A). The intracranial vessels were normal at the CT angiogram (CTA). The clinical course was complicated by a focal epileptic seizure with subsequent generalization; therefore, an antiepileptic therapy with carbamazepine was started.

To investigate the causes of intestinal obstruction, several diagnostic assessments were conducted. He underwent an abdominal CT and MRI and a colonoscopy to rule out expansive and infiltrative lesions, and total body positron emission tomography (PET) and a periumbilical fat biopsy were performed to exclude systemic vasculitis and amyloidosis, respectively. Therefore, a diagnosis of chronic intestinal pseudo-obstruction (IPO) was formulated. The patient was treated with pro-kinetic drugs and supported with parenteral nutrition, with progressive clinical improvement and restoration of intestinal transit. Lab tests also showed high serum lactate (1.7 mmol/l, normal range 0.0–1.3 mmol/l), hyponatremia and hypokalemia, probably due to inappropriate secretion of antidiuretic hormone syndrome (SIADH) caused by the carbamazepine therapy. The electrolytic disturbance was corrected, and carbamazepine was replaced with levetiracetam without neurological clinical improvement.

**Figure 2:**
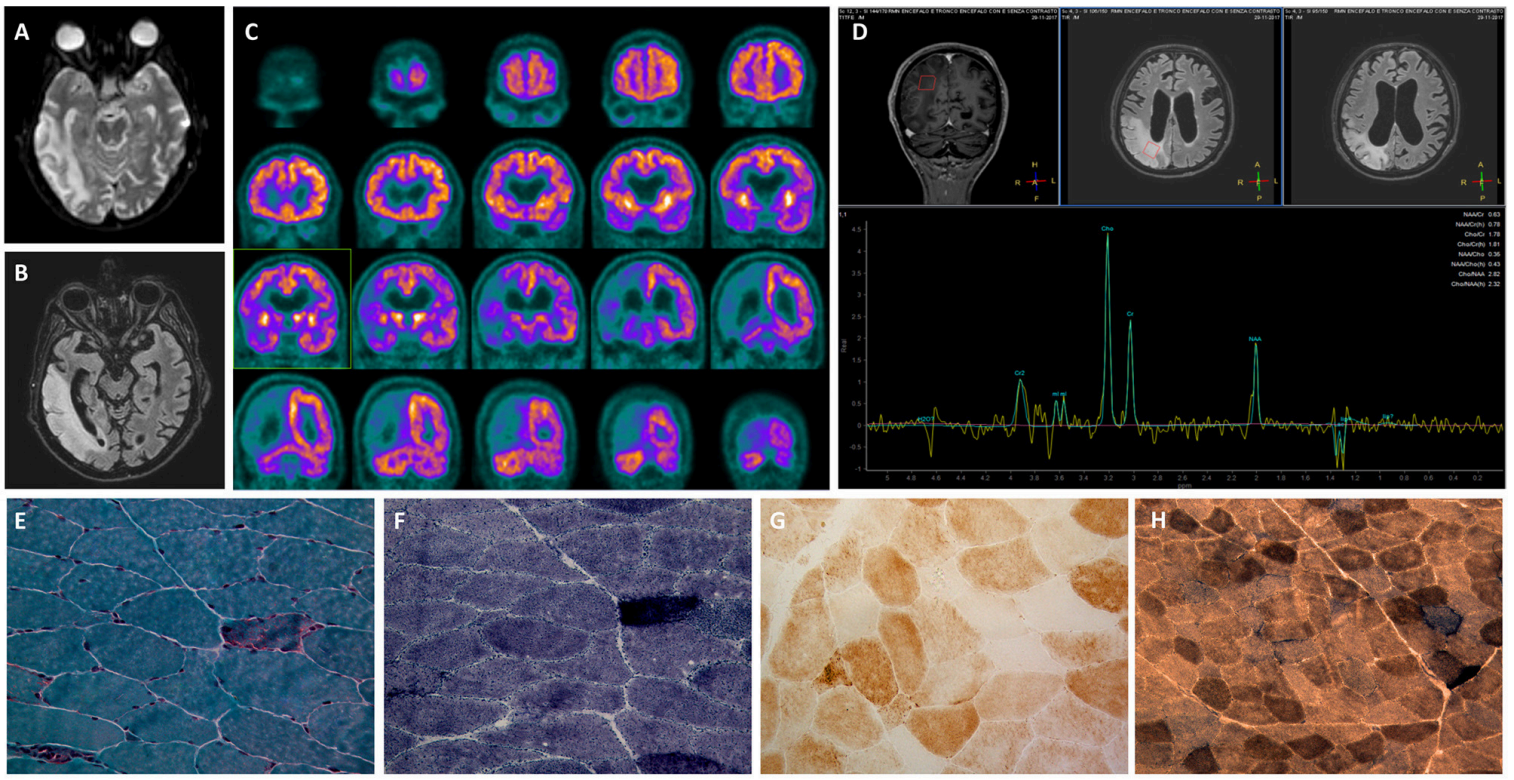
Diagnostic assessment of MELAS syndrome. **(A–B)** Cortical diffusion-weighted imaging abnormalities in the right temporal, parietal and occipital lobes with corresponding fluid attenuated inversion recovery hyperintensity. **(C)** Brain positron emission tomography (PET) revealed a severe reduction in cortical glucose metabolism in the posterior right hemisphere. **(D)** MR spectroscopy (MRS) showed elevation of the lactate peak within the abnormal lesion. **(E–H)** Muscle biopsies. **(E)** Modified Gomori Trichrome stain (40X) showing a typical Ragged Red muscle fiber (RRF) containing mitochondrial hyperproliferation. **(F)** Succinate dehydrogenase (SDH) stain (40X) confirming mitochondrial hyperproliferation in the same fiber. **(G)** Cytochrome C oxidase (COX) stain (40X) demonstrating muscle cells with decreased mitochondrial activity. **(H)** COX/SDH combo reaction (20X) showing diffuse fibers with COX deficiency and SDH positivity scattered in the muscle.

He was admitted to our Neurology Department for persistence of confusion and development of left arm clumsiness and stiffness. The brain MRI was repeated and showed evolution of the right hemispheric lesion, which extended to the parietal lobe and the anterior and medial parts of the temporal lobe and involved the subcortical white matter and cortex (Figures 1B, 2A,B). The electroencephalogram (EEG) showed slow persistent activity and periodic lateralized epileptiform discharges (PLEDs) in the right hemisphere, whereas the brain PET revealed a severe reduction in cortical glucose metabolism in the posterior right hemisphere (Figure 2C). Therefore, a metabolic etiology of the disturbance was suspected. To confirm this hypothesis, the patient underwent MR spectroscopy (MRS) and demonstrated elevation of the lactate peak within the abnormal lesion (Figure 2D), a muscle biopsy that was consistent with mitochondrial myopathy (Figures 2E–H) and genetic testing, which revealed the presence of a mitochondrial DNA mutation (m.3243A>G) (heteroplasmy 13.1%) in the MT-TL1 gene encoding the leucine transfer RNA. A diagnosis of MELAS was formulated, and therapy with oral arginine, ubidecarenone and riboflavin was administered to the patient.

The genetic analysis was extended to his sister, nephews and two first grade cousins; the family tree is shown in Supplementary Figure 1. Four of these family members were positive for heteroplasmy and were asymptomatic. The audiological examination revealed bilateral sensorineural hearing loss.

Three months after hospital discharge, the patient presented with a new onset of an acute confusional state, visual illusion in the right visual field and severe frontal headache. At the neurological examination, the patient appeared confused, slowed down and disoriented. The confrontation visual field test showed right superior quadrantopsia together with the previous left hemianopsia. He also had face-blindness, visual agnosia, left upper arm apraxia and mild anomic aphasia. The lab tests showed increased serum lactate (2.9 mmol/l; normal range 0.0–1.3 mmol/l). A partial resolution of the previous right cortical lesion and the presence of a new cortical DWI abnormality in the left medial temporal and occipital lobes was observed at the brain MRI (Figure 1C, lower part), revealing a new stroke-like episode. Additionally, the Fluid Attenuated Recovery (FLAIR) sequences identified a marked and greater cortical atrophy with increased ventricular sizes (Figure 1C, upper part). To counter the vasogenic edema resulting from blood-brain barrier dysfunction due to mitochondrial microangiopathy, the patient received intramuscular corticosteroids (dexamethasone 8 mg), but the treatment was prematurely stopped due to onset of drug-induced diabetes mellitus, and insulin therapy was started.

**Figure 3:**
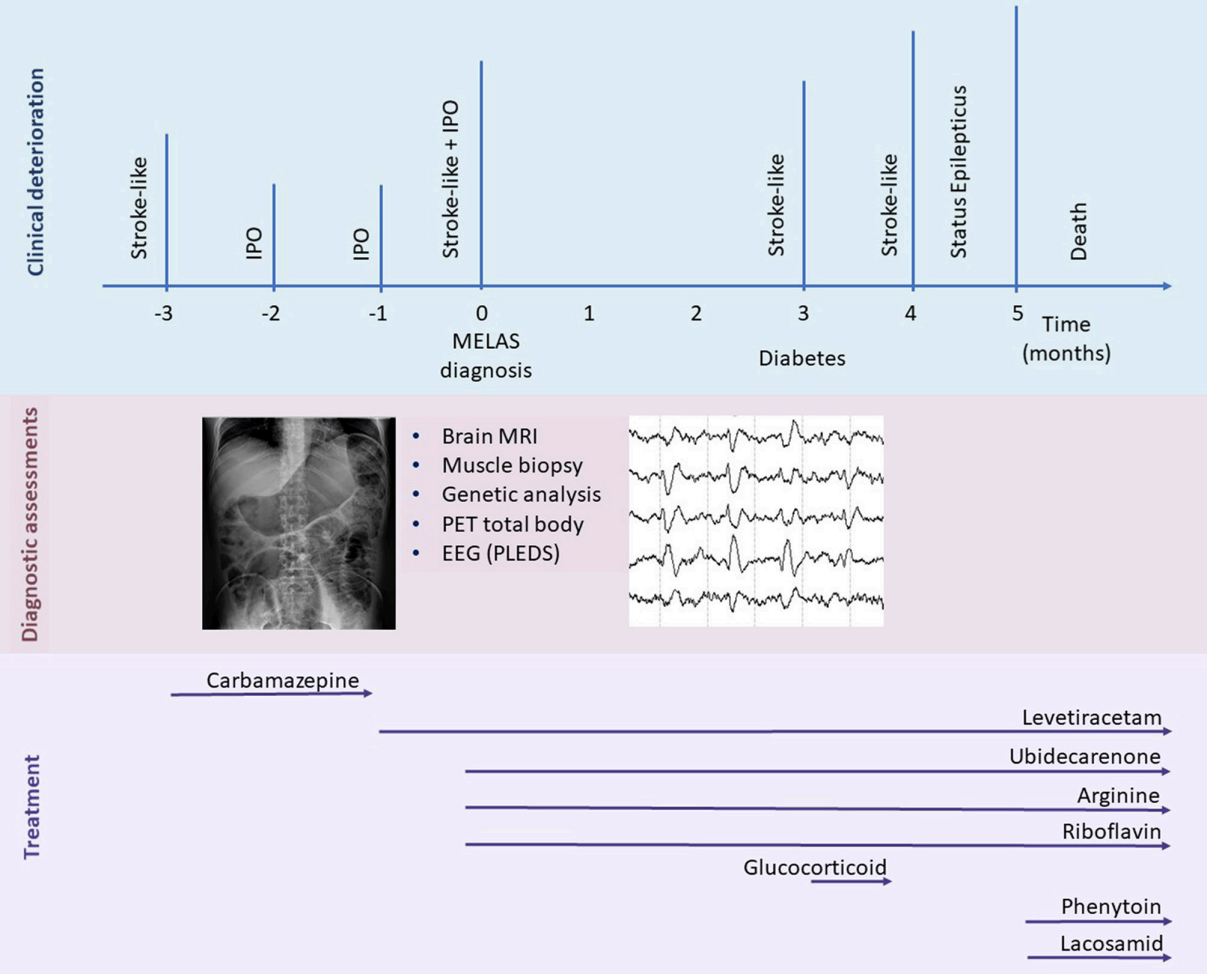
Timeline representing the clinical disease course. Major clinical episodes are shown in the first line, and the diagnostic assessments performed and treatments are reported in the second and third lines, respectively. IPO, intestinal pseudo-obstruction; MELAS, mitochondrial encephalomyopathy, lactic acidosis, and stroke-like episodes; PLED, period lateralized epileptiform discharges.

In the following month, the patient developed a rapidly progressive ideomotor decline; the patient had spatial and temporal disorientation, psychomotor agitation, speech disturbance with confabulation and cortical-blindness. A new left lateral temporal and occipital lesion was identified on brain MRI (not available); oral arginine therapy was increased, and intravenous L-arginine was administered. During hospitalization, the patient suffered again from acute IPO and was treated conservatively. He also manifested a non-convulsive epileptic status. To achieve seizure control, lacosamide, phenytoin, and clobazam were progressively added to the levetiracetam. Despite therapeutic implementation, the patient did not recover, and he died one month later (Figure 3).

## Discussion

We describe a late-onset MELAS syndrome case due to the classic m.3243A>G mutation that presented with relapsing IPO episodes. The patient suffered from several stroke-like episodes in rapid sequence, which contributed to a rapid and relentless ideomotor deterioration and carried him to a fatal ending.

Some elements make this patient atypical. Firstly, he presented for medical attention at the age of 50 years and was almost completely asymptomatic until the first stroke-like episode occurred. Childhood represents the typical age of onset of MELAS patients, with onset occurring before or at 20 years of age in 65–76% of cases (8). Only a few cases (1–6%) are older than 40 years of age at presentation. Second, he presented a particularly aggressive clinical course and died only a few months after onset of the disease. In contrast, usually the juvenile form is associated with a more aggressive course and deterioration than the adult form (1), and juvenile patients commonly have faster disease progression and higher mortality than adults (1). This disease course probably depends on two aspects: the cells of the organism need higher amounts of energy during childhood due to system development, and an early presentation of symptoms is more often associated with a higher mutation load in the mtDNA (9). Despite the high percentage of relatives carrying the disease-causative mutation, our patient is the only affected member of his family to date. This result could be partially due to the heteroplasmy phenomenon and the younger ages of his relatives, who may present later in time; nevertheless, other mechanisms may be involved in determining the clinical presentation.

One of the main clinical features of our patient was the occurrence of relapsing IPO episodes concomitant with his first clinical presentation. Although it is mainly reported in association with MNGIE, a paralytic ileus can also represent a severe complication in MELAS-affected patients and seems to be relatively frequent in patients harboring the m.3243A>G mutation (10). Furthermore, gastrointestinal involvement is more severe in MELAS patients with the m.3243A>G mutation than in MNGIE patients (11). Chronic IPO with any cause affects 0.9 out of 100000 people (12), whereas the minimum prevalence of IPO in patients with the m.3243A>G mutation is 0.53 per 100000 (11). Sekino et al. found a 40% prevalence of IPO in MELAS-affected patients and reported a lower age at onset and at diagnosis in patients with IPO than in those without IPO in their cohort (13). In a large observational study of 215 patients harboring the m.3243A>G mutation, 13% of the patients presented acutely with IPO (11).

The pathophysiological mechanisms underlying gastrointestinal dysmotility in mitochondrial disorders are not completely understood and most likely involve myenteric plexus neuropathy and visceral myopathy. The identification of cytochrome c oxidase (COX) deficiency within the smooth muscle layers of the gastrointestinal tract in two patients carrying the classic mutation in the MT-TL1 gene supports the evidence of visceral myopathy in mitochondrial patients (14). Moreover, numerous and enlarged mitochondria in smooth muscle cells and the cytoplasm of ganglion cells were found in a segment of resected ileum and colon from a MELAS-affected patient (5). These findings suggest that mitochondrial dysfunction and energy production impairment may be the basis for the gastrointestinal symptoms in these patients, similar to the effects in the brain during stroke-like episodes.

Stroke-like episodes manifest with an acute onset neurological deficit that is sometimes accompanied by headache, vomiting and seizures. Since gastrointestinal disturbance and stroke-like episodes share common cellular and molecular substrates, their concomitant occurrence is not unlikely.

We conducted a comprehensive literature search of the MEDLINE database from 2000 to September 2018 and retrieved 11 studies and case reports describing the occurrence of IPO in MELAS syndrome patients (Table 1). The terms used for the search were as follows: (MELAS OR mitochondrial encephalomyopathy, lactic acidosis, and stroke-like episodes OR mitochondrial diseases) AND (intestinal pseudo obstruction OR gastrointestinal involvement).

**Table 1:** Case reports of intestinal pseudo-obstruction in MELAS syndrome.

**Reference author, date**	**IPO after MELAS diagnosis**	**Concomitant IPO and MELAS diagnosis**	**GI involvement before MELAS diagnosis**	**IPO treatment and outcome**	**Other comments**
Sekino et al. (13)	6	2	1	Conservative 2 deceased from cardiomyopathy, 1 deceased from cerebral infarction, 1 deceased from epilepsy and aspiration pneumonia	–
Ng et al. (11)	–	8	1	3 surgery 4 deceased from aspiration pneumonia	8 concomitant IPO and stroke-like
Suzuki (15)	3	–	2	Conservative Died from aspiration pneumonia	–
Betts et al. (14)	2	–	1	Conservative	–
Primiano et al. (16)	–	–	1	Prucalopride Recovery	–
Chang et al. (5)	1	–	1	Laparoscopy	MELAS/MNGIE overlap
Chiyonobu (17)	1	–	–	Conservative	Concomitant IPO and status epilepticus
Nakae (18)	1	–	–	Conservative Recovery	Concomitant IPO and status epilepticus
Seessle (19)	1	–	–	Subtotal colectomy, ileorectostomy Recovery	–
García-Velasco et al. (20)	1	–	1	Conservative Recovery	Concomitant IPO and stroke-like
Muehlenberg et al. (21)	–	1	1	Gastrectomy for perforation Survival	Concomitant diagnosis and stomach perforation

*IPO, intestinal pseudo-obstruction; MELAS, mitochondrial encephalomyopathy, lactic acidosis, and stroke-like episodes. Sixteen cases presented with IPO occurring after the MELAS diagnosis, concomitant IPO and MELAS diagnosis were reported in 11 patients (see Comments for specimens; e.g., concomitant IPO and stroke-like episode), and general gastro-enteric symptoms were reported before the MELAS diagnosis in nine patients. Conservative treatments consisted of laxative agents, antidiarrheal drugs, antiflatulence agents, mosapride, dimethicone, pantothenic acid, daikenchuto (Chinese herbal medicine), neostigmine or distigmine, domperidone, mosapride citrate, butyric acid bacteria, sodium picosulfate, prostaglandin F2 alpha, pantothenic acid, dioctyl sodium sulfosuccinate or eritromycine*.

Garcia-Velasco and colleagues described the case of a newly diagnosed patient who presented with simultaneous stroke-like and IPO symptoms (20). In a recent observational study aimed at determining the prevalence of IPO in m.3243A>G-related mitochondrial disease manifesting with IPO, 8 of 30 patients developed a stroke-like episode simultaneously with IPO (11). In the same cohort, relapsing IPO episodes preceded the first stroke-like episodes by approximately 20 years (11). To the best of our knowledge, gastrointestinal symptoms, especially IPO, led to the diagnosis of MELAS in six patients (11, 13, 21). We summarized the previous reports of MELAS syndrome patients with the occurrence of IPO in Table 1.

In our case, the occurrence of IPO led us to reconsider the previous ischemic stroke diagnosis as a stroke-like episode and to postulate a diagnosis of MELAS. Additionally, the brain MRI performed during acute presentation of IPO showed an evolution of the previously observed lesion. Since that time, several stroke-like episodes had occurred over a short time period, as if the first stroke-like episode had triggered a cascade of events and led to progressive, rapid, and unstoppable multiorgan metabolic failure. Furthermore, the patient experienced a rapidly progressive cognitive decline, which carried him to death, as if the disease had assumed a neurodegenerative trend until reaching a point of no return. Finally, our patient was almost completely asymptomatic until the age of 50 years, when the first stroke-like episode occurred.

In the case described here, the manifestation of IPO developed over a period of time that was almost concomitant with the appearance of the first stroke-like episode. Precisely determining that one of the two symptoms was the precipitating cause of the other is difficult. The gastrointestinal dysfunction accompanied the progressive deterioration from the first episode to the final worsening that led to the exitus. We speculated that the energy balance of cells of our patient, even if unstable, remained well-compensated for a long time until a single event (e.g., the first stroke-like episode or the acute IPO) overcame a threshold and induced the breakdown of the energetic system, prompting multiorgan deterioration. Indeed, he developed a severe ideomotor decline that was entirely comparable to a neurodegenerative process; he had a focal epileptic status, manifested diabetes and suffered from chronic relapsing IPO. The gastrointestinal disturbance could have played a determinant role in this frail scenario as both a causative agent and an epiphenomenon of a fatal disease. A centrally mediated mechanism called the “neurogastrointestinal crisis” has also been hypothesized to explain the concomitant occurrence of severe stroke-like episodes and IPO (11).

Recently, the prognostic role of IPO in mitochondrial disorders has been recognized, in particular in patients carrying the m.3243A>G mutation (7). Indeed, IPO is most frequently observed in patients with multiorgan involvement and a higher disease burden (11).

However, together with positive urine heteroplasmy, a low mass body index (BMI) and a history of cardiomyopathy, stroke-like episodes represent the strongest predictor for developing IPO (Hazard Ratio 2.93, *p* = 0.01) (11).

Treatment of stroke-like episodes includes intravenous L-arginine in the acute phase and oral L-arginine, riboflavin, and idebenone (coenzyme Q is able to cross the blood brain barrier) in the interictal phases (22). High-dose intravenous corticosteroids have been used to reduce the vasogenic edema of stroke-like lesions (23, 24). Management of the paralytic ileus should be based on conservative treatment, since surgical intervention has been associated with a poor prognosis in patients with mitochondrial diseases. In particular, a successful outcome has been obtained with prucalopride (16). Unfortunately, medical therapy was ineffective in our patient due to the elevated disease burden.

In conclusion, IPO as a manifestation of mitochondriopathy is a misdiagnosed and underdiagnosed entity. Additionally, a longstanding history of gastrointestinal dysmotility is often underestimated before acute presentation. Since stroke-like episodes and intestinal dysmotility can interfere with each other and may predict a worse clinical outcome, clinicians should be aware of the possibility of an even worse prognosis.

Early detection of chronic gastroenterological disturbances in mitochondriopathies may prompt precocious identification of neurological comorbidities, leading to appropriate therapeutic strategies and improved clinical outcomes.

## Ethics Statement

The case report has been performed in accordance with the ethical standards laid down in the 1964 Declaration of Helsinki and its later amendments. Informed written consent was obtained from the participant for the publication of this manuscript.

## Author Contributions

All authors took care of patient management and made decisions about patient treatment. DG and EM conceived the idea, revised the literature, and wrote the manuscript. FM, DV, and MM performed a critical revision of the manuscript for important intellectual content. EA, RB, and IF acquired the clinical data. DP and DR performed the genetic analysis. FT analyzed and interpreted the brain imaging. LP and MS analyzed the muscle biopsy. RB, EM, FM, and AG collected the clinical data and contributed to manuscript preparation. NB, GC, SC, and AG contributed to manuscript revision and read and approved the submitted version.

### Conflict of Interest Statement

The authors declare that the research was conducted in the absence of any commercial or financial relationships that could be construed as a potential conflict of interest.

## Supplementary Material

Supplementary Figure 1 Patient's family pedigree showing transmission of 3243A>G through four generations.
