# Supplementary figures and images for: Can Intestinal Pseudo-Obstruction Drive Recurrent Stroke-Like Episodes in Late-Onset MELAS Syndrome? A Case Report and Review of the Literature
Source: Front Neurol. 2019 Jan 31;10:38. doi: 10.3389/fneur.2019.00038 (PMC6365425; doi:10.3389/fneur.2019.00038)

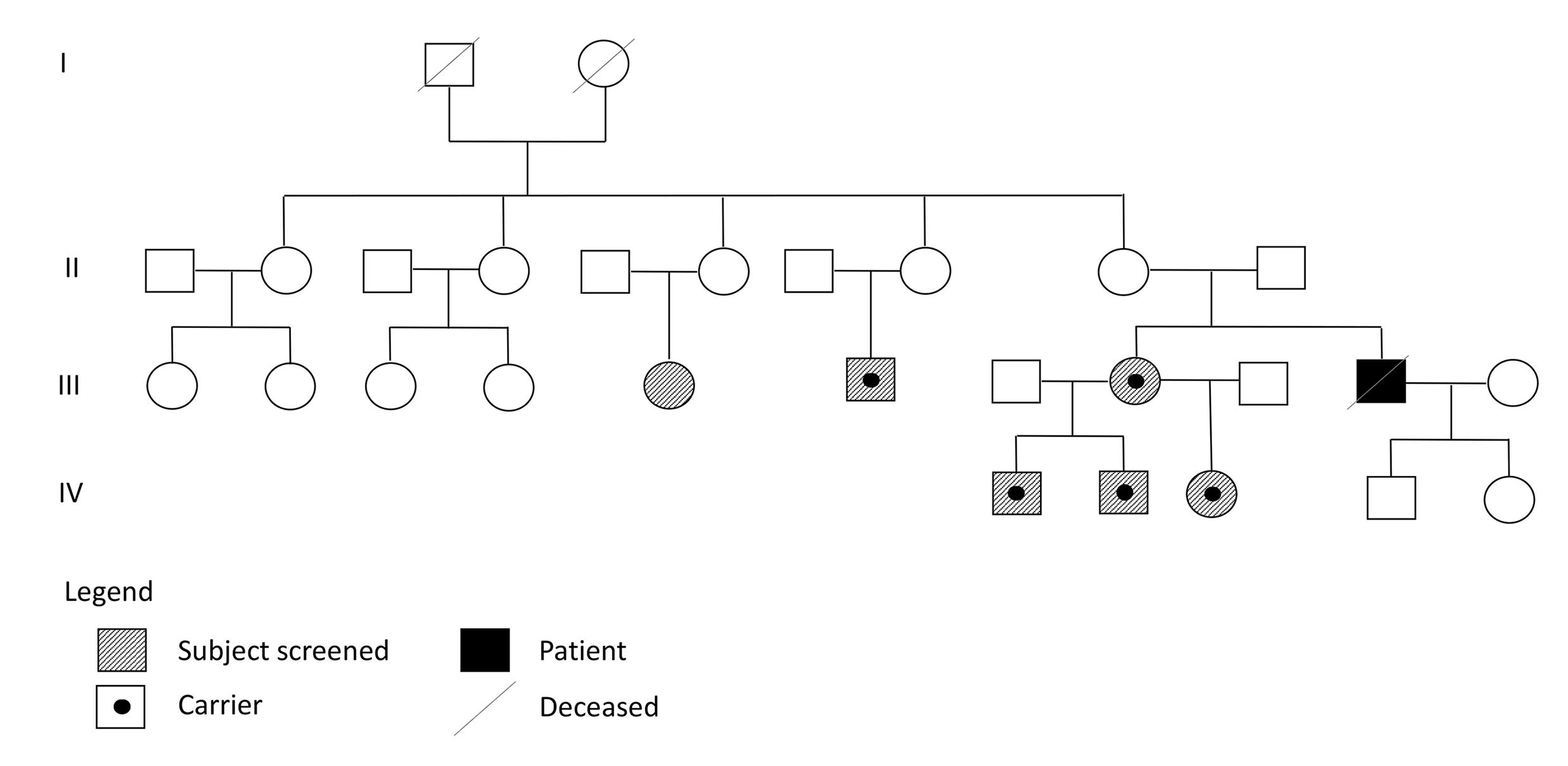

Supplement: Supplementary Figure 1 — Patient's family pedigree showing transmission of 3243A>G through four generations. [file Image_1.JPEG]
